# HaMStR: Profile hidden markov model based search for orthologs in ESTs

**DOI:** 10.1186/1471-2148-9-157

**Published:** 2009-07-08

**Authors:** Ingo Ebersberger, Sascha Strauss, Arndt von Haeseler

**Affiliations:** 1 Center for Integrative Bioinformatics Vienna (CIBIV), Max F. Perutz Laboratories, Vienna, Austria; 2 University of Vienna, Vienna, Austria; 3 Medical University of Vienna, Vienna, Austria; 4 University of Veterinary Medicine, Vienna, Austria

## Abstract

**Background:**

EST sequencing is a versatile approach for rapidly gathering protein coding sequences. They provide direct access to an organism's gene repertoire bypassing the still error-prone procedure of gene prediction from genomic data. Therefore, ESTs are often the only source for biological sequence data from taxa outside mainstream interest. The widespread use of ESTs in evolutionary studies and particularly in molecular systematics studies is still hindered by the lack of efficient and reliable approaches for automated ortholog predictions in ESTs. Existing methods either depend on a known species tree or cannot cope with redundancy in EST data.

**Results:**

We present a novel approach (HaMStR) to mine EST data for the presence of orthologs to a curated set of genes. HaMStR combines a profile Hidden Markov Model search and a subsequent BLAST search to extend existing ortholog cluster with sequences from further taxa. We show that the HaMStR results are consistent with those obtained with existing orthology prediction methods that require completely sequenced genomes. A case study on the phylogeny of 35 fungal taxa illustrates that HaMStR is well suited to compile informative data sets for phylogenomic studies from ESTs and protein sequence data.

**Conclusion:**

HaMStR extends in a standardized manner a pre-defined set of orthologs with ESTs from further taxa. In the same fashion HaMStR can be applied to protein sequence data, and thus provides a comprehensive approach to compile ortholog cluster from any protein coding data. The resulting orthology predictions serve as the data basis for a variety of evolutionary studies. Here, we have demonstrated the application of HaMStR in a molecular systematics study. However, we envision that studies tracing the evolutionary fate of individual genes or functional complexes of genes will greatly benefit from HaMStR orthology predictions as well.

## Background

The amount of protein-coding DNA sequences in the public data bases is steadily increasing. This data is mainly generated by the sequencing and annotation of entire genomes and by numerous EST sequencing projects. Approaches to resolve the evolutionary relationships of eukaryotes on a molecular basis -frequently referred to as molecular systematics- particularly benefit from this data. Recent studies on the evolution of metazoans and fungi present trees with 40 to 77 taxa, reconstructed from more than 140 genes [1-6]. Still, these studies consider only a small fraction of the data available. For example, as of May 2008 dbEST contains 714 eukaryotic taxa with more than 2.000 ESTs each, and 394 taxa have more than 10,000 ESTs . Despite the potential value of ESTs especially for molecular systematics [7] this data has rarely been used in phylogenetic studies so far. This is due to the fact that ESTs are redundant, short and of low sequence quality. Plenty of solutions exist to improve data quality by cleaning and clustering of ESTs sequences prior to analysis [8-10]. However, annotating ESTs and more importantly inferring their relationships to known genes in other taxa is still problematic.

For most molecular systematics approaches only sequences are admissible for which the orthology [11] or co-orthology [12] to the other sequences in the dataset is known, or at least reasonable to assume [6] (but see [13]). A variety of tools exist to define groups of orthologous proteins. Among these, phylogeny-based programs, such as RIO [14], reveal the lowest fraction of false positives in a comparison of 10 programs for orthology/homology inference [15]. It is obvious that methods depending on a known species tree are not applicable if the aim is to infer the phylogenetic relationships of taxa. However, more recent approaches have found solutions to overcome this strict dependence on a known species tree, *e.g*., [16]. The reciprocal best BLAST hit criterion (RBH) between pairs of sequences from two taxa, is frequently used to infer orthology when the species tree is unknown. Orthology predictions from the RBH method have a very low false positive rate [15]. However, ortholog groups are ultimately confined to two sequences. Thus, co-orthologs resulting from gene duplications after the separation of the compared taxa are missed. Several variations exist to extend RBH ortholog groups to allow for the addition of co-orthologs [12] and/or to extend the prediction to more than two taxa [17-19].

Orthology prediction methods based on the RBH criterion have been designed for, and successfully applied to, inference of ortholog groups for taxa for which the entire genome has been sequenced. Their application to EST based data sets, however, is not straightforward. Incomplete representation of a species' gene set, especially in small EST projects, depreciates a reciprocal best BLAST hit as an indicator of orthology. Moreover, the coding sequence of a gene can be tagged by two or more non-overlapping ESTs. Methods such as InParanoid [12] or orthoMCL [18] will in most cases add only one of the ESTs to the ortholog cluster. Both programs allow for more than one sequence per species in an ortholog cluster, only when the intra-specific similarity for the sequences in the cluster is higher than the sequence similarity between species. However, the intra-specific similarity between non-overlapping ESTs derived from the same gene is approximately that of two randomly chosen and unrelated sequences.

To use EST data in molecular systematics, sequences have been initially BLASTed against a protein sequence database, *e.g*., the non-redundant protein database at NCBI, and the highest scoring hits are taken to tentatively annotate the EST sequences [20]. Post-processing of the BLAST results to infer the orthology status of the query-hit pair ranged then from the application of simple e-value cutoffs, *e.g*., [21], to more complex procedures including Markov Clustering and visual inspection of individual sequence trees [2]. Problematic to these approaches is that they unduly substitute e-values of a BLAST result with evolutionary relationships, involve too many human interactions to be routinely applied, or suffer from the conceptual problem of Markov Clustering when applied to non-overlapping sequence fragments representing the same protein.

Here we present a novel approach, HaMStR, to extend predefined groups of orthologous genes derived from taxa, whose genomes have been fully sequenced with data from further taxa. We show that the joint application of a profile Hidden Markov Model (pHMM) based similarity search and a subsequent re-BLAST of the hit sequences against a reference proteome identifies candidate orthologs. HaMStR exhibits a very low false positive rate and good sensitivity. The resulting collection of orthologous sequences provides an excellent basis for phylogeny reconstruction.

## Results

### Algorithm

#### Step 1 Defining a Gene Set for the Ortholog Search

##### 1.1 Generation of core-orthologs

As input we introduce the **primer-taxa (set) **where each taxon is completely sequenced and where the phylogeny of the primer-taxa is undisputed. Standard orthology prediction tools such as InParanoid [12] or orthoMCL [18] can be applied to identify genes with orthologs present in all primer taxa, the so called **core-ortholog **groups. For this study we compute orthologs for each pair of taxa with InParanoid [12]. The pair-wise orthology predictions are subsequently extended to include all primer taxa by using a criterion of transitive closure (InParanoid-TC). We end up with a collection of core-orthologs where in each individual core-ortholog every primer taxon is represented exactly once. Details about this procedure are provided online (see Additional file 1).

##### 1.2 Generation of profile Hidden Markov Models

For each sequence cluster in the core-orthologs, the sequences are aligned using MAFFT [22] with the options --*maxiterate 1000 *and --*localpair*. The resulting multiple sequence alignments, comprising the *n *sequences from the primer-taxa are then converted into a profile Hidden Markov Model (pHMM) [23]. The programs *hmmbuild *and *hmmcalibrate *from the HMMER package  are used for building, training and calibrating the pHMMs. Each core-ortholog is now represented by a pHMM.

#### Step 2 Extension of core-orthologs

We now extend the core-orthologs with data from additional taxa, the query-taxa. As data may serve translated ESTs, or protein sequences inferred from either complete or partially sequenced genomes.

##### 2.1 pHMM search

We use a fast implementation of the *hmmsearch *algorithm  to search protein sequence data from the query-taxon for matches to the individual pHMMs. If ESTs are the data for the query taxa, the individual ESTs are translated in all six reading frames prior to the search.

##### 2.2 Re-BLAST and orthology prediction

**Figure 1:**
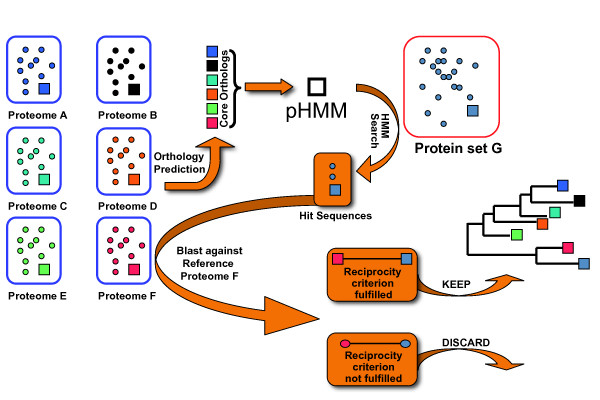
**Workflow of the HaMStR approach**. Standard orthology prediction tools are used to identify orthologous groups, the so called core-orthologs, for a set of completely sequenced primer taxa (Proteome A - F). The sequences in a core-ortholog are aligned and converted into a profile HMM (pHMM). A compilation of protein sequences or translated ESTs from a taxon not included in the primer-taxa (Protein set G) is searched for hits with the pHMM. The resulting candidates display features that are characteristic for the protein modelled by the pHMM. To determine the orthology status of the candidates, we introduce a reciprocity criterion. Each candidate is compared by BLASTP with the proteome of one of the primer-taxa, the so-called reference-taxon (Proteome F). If the best BLASTP hit sequence from the reference taxon corresponds to the protein that contributed to the pHMM, the candidate is called candidate-ortholog, else it is discarded.

To determine the orthology status of the *hmmsearch *hits, we use a reciprocity criterion (Figure 1). Each hit is compared by BLASTP [24] to the proteome of one of the primer-taxa, the so-called reference-taxon (Proteome F in Figure 1). Ideally, the reference-taxon should be the closest related primer-taxon to the query-taxon. If the protein of the reference taxon that contributed to the pHMM provides the best BLASTP hit, then the *hmmsearch *hit is added to the corresponding core-ortholog. Otherwise, it is discarded. Please note that the reciprocity criterion is also fulfilled when the reference protein is among the lower ranking BLASTP hits, but has the same score as the top listed hit in the BLASTP output.

##### 2.3 Post-processing of ESTs

To account for possible frame shifts caused by sequencing errors in ESTs we use *genewise *[25] to generate a codon-alignment for the EST and the protein sequence of the reference-taxon. This alignment determines the coding part together with the reading frame in the EST.

### Testing

We next test the versatility of HaMStR. The data for this step are summarized in Table 1.

**Table 1:** Overview of the data and data sources used in this study

**Proteins**	
**InParanoid**^**a**^	*Homo sapiens*
	*Monodelphis domestica*
	*Ciona intestinalis*
	*Drosophila melanogaster*
	*Caenorhabditis elegans*
	*Saccharomyces cerevisiae*
	*Debaryomyces hansenii*
	*Kluyveromyces lactis*
	*Candida glabrata*
	*Yarrowia lipolytica*
**Broad Institute**^**b**^	*Uncinocarpus reesii*
	*Stagonospora nodorum*
	*Chaetomium globosum*
	*Clavispora lusitaniae (Candida lusitaniae)*
	*Pichia guillermondii (Candida guillermondii)*
	*Candida tropicalis*
	*Candida albicans*
**UniProt**^**c**^	*Aspergillus fumigatus*
	*Aspergillus terreus*
	*Aspergillus oryzae*
	*Ashbya gossypii*
**Genoscope**^**d**^	*Podospora anserina*

**ESTs**	

**dbEST**^**e**^	*Ustilago maydis *(39308)
	*Cryptococcus neoformans *(59041)
	*Phanerochaete chrysosporium *(13189)
	*Coprinopsis cinerea *(15715)
	*Schizosaccharomyces pombe *(8123)
	*Ajellomyces capsulatus *(26389)
	*Neurospora crassa *(20089)
	*Trichoderma atroviride *(1656)
	*Trichoderma asperellum *(1882)
	*Trichoderma harzianum *(12165)
	*Fusarium oxysporum *(9248)
	*Bortrytis cinerea *(10982)
	*Sclerotinia sclerotiorum *(1494)
	*Fusarium graminearum *(6678)
**TGI**^**f**^	*Coccidioides immitis *(9312)
	*Aspergillus nidulans *(13100)
	*Fusarium verticillioides *(11126)
	*Magnaporthe grisea *(20890)

^a^

^b^

^c^

^d ^

^e ^. The numbers of ESTs per organism are given in parenthesis.

^f ^. The numbers of tentative consensus sequences are given in parenthesis.

We analyze three scenarios. In all cases we wanted to detect orthologs in the human proteome, where the primer-taxa set consists of opossum (*Monodelphis domestica*), sea squirt (*Ciona intestinalis*), fruit fly (*Drosophila melanogaster*), worm (*Caenorhabditis elegans*), and a fungus (*Saccharomyces cerevisiae*). The corresponding core-ortholog set (PoP) represents 994 genes.

In the first scenario, we carried out *step 1 *of HaMStR and extracted the opossum proteins from the 994 core-orthologs. Then we used InParanoid to infer the corresponding orthologs in the human proteome. InParanoid identified 979 human-opossum ortholog clusters. For 15 opossum genes InParanoid did not find a human ortholog.

In the second scenario, we applied *step 2 *of HaMStR with the opossum as reference-taxon instead of using InParanoid. If HaMStR predicts more than one ortholog, we use ClustalW [26] to align all candidates individually to the opossum protein in the corresponding core-ortholog. The human proteins are then ranked according to their alignment score. HaMStR predicted 976 human orthologs. For the remaining 18 core-orthologs no human counterpart was found.

For 972 core-orthologs the top ranking HaMStR ortholog is the same as those found with InParanoid (Table 2). Four human genes that have been found with InParanoid were not detected in *step 2 *of HaMStR. In all instances, InParanoid identified several proteins as co-orthologs to the human gene. However, only one of these opossum sequences was used to train the corresponding pHMMs. During the re-BLAST (c.f. 2.2), a different co-ortholog from the same InParanoid-ortholog cluster was obtained as the best BLASTP hit. Thus, the reciprocity criterion was not fulfilled and HaMStR did not make an orthology prediction.

**Table 2:** Ortholog search for 994 evolutionary conserved genes in the human proteome

	**HaMStR**	**InParanoid**
**orthologs predicted**^**a**^	976	979
**identical**	972	972
**different**	-	4
	1	-
	3	3
**no prediction**	14	14
	4	-
	-	1

**total**	994	994

^a ^*different *denotes those instances where either both programs predict a different human protein as ortholog, or where an ortholog is predicted only by one program.

One human gene was found with HaMStR but not with InParanoid.

For three core-orthologs InParanoid and HaMStR suggested different human proteins. For the core-ortholog represented by the opossum protein ENSMODP00000017416 InParanoid and HaMStR suggest as orthologs the proteins ENSP00000363169 and ENSP00000371363, respectively. Both proteins are InParanoid co-orthologs with respect to *Ciona *and with respect to fishes. It appears plausible, that the gene duplication giving rise to the protein pair ENSP00000363169/ENSP00000371363 occurred recently, and presumably around the same time as the split of the human and opossum lineages. This view is supported by the observation that the BLAST score (InParanoid) of ENSMODP0000017416/ENSP00000363169 exceeds the score of ENSMODP0000017416/ENSP00000371363, whereas the scores of the global alignment (HaMStR) produce the opposite result. Moreover, orthoMCL [18] groups both human proteins together with the opossum protein in the same ortholog cluster. A similar scenario applies to the second case where InParanoid and HaMStR disagree (Table 2). In the third case, ENSP00000222402 is according to HaMStR ortholog to the *M. domestica *protein ENSMODP00000011077 whereas it is ortholog to ENSMODP00000017386 according to InParanoid. The latter protein, however, is present only as a truncated sequence in our set of opossum proteins, lacking 8 amino acids. This truncation results in the same BLASTP score in the re-BLAST (c.f. 2.2) between ENSP00000222402 and both opossum proteins. Again, orthoMCL groups these proteins into the same ortholog cluster.

In summary, the HaMStR strategy is suitable for identifying orthologs for a pre-defined gene set. The results are consistent with those obtained with one of the most efficient existing orthology prediction programs. For 168 core-orthologs, more than 1 human protein was assigned as a putative ortholog. A detailed description of these lower ranking hits is given in the online supplementary material (see Additional file 2).

The HaMStR approach is efficient in identifying orthologs when the entire sequence of the query protein is known. In scenario 3, we assess the performance of HaMStR when using translated ESTs. In many molecular systematics studies, EST projects are typically small [21,27,7]. To assess the performance of HaMStR in such cases, we did not cluster the ESTs. Each single EST is now an input for HaMStR.

As a typical example we selected 32,647 ESTs derived from human chromosome 2  according to the following criteria: A Blat search [28] with each EST against the human genome sequence must obtain a best hit on chromosome 2 covering at least 90% of the EST. Moreover, the best Blat hit must be located between the annotated start, and the end of the transcript of an annotated gene (Ensembl database; ), and must overlap with a coding exon. The 32,647 ESTs (hchr2-ESTs) map to 1,106 genes. From the analyses in scenario 1 and 2 we know that only 81 of these are represented by core-orthologs in the PoP primer-taxa set. This set of 81 human chromosome 2 genes is tagged by 6,288 ESTs (19%).

From the 32,647 hchr2-ESTs that served as HaMStR input, 29,293 were not assigned to any of the 994 core-orthologs (Table 3). The remaining 3,354 ESTs are highly enriched (97%) with ESTs tagging the 81 genes that are represented in the PoP set. More precisely, 3,243 ESTs were assigned correctly to 72 core-orthologs. The remaining 3% were assigned to two core-orthologs for which the human gene is not located on chromosome 2.

**Table 3:** HaMStR ortholog search in human chromosome 2 ESTs

	**Genes**	**ESTs**
**Total**^**a**^	1106	32647
**Max. no. of hits**^**b**^	81	6288
**Not annotated**	1032	29293
**Orthologs predicted**	74	3354
**Orthologs (id**^c^)	72	3243
**Orthologs (diff^c^)**	2	111
**Orthologs (missed^c^)**	9	389
**False positive rate**	3%	3%
**Sensitivity**	89%	55%

^a ^*Total *denotes the number genes/ESTs in the chr2-EST data.

^b ^Intersection of the genes represented in the chr2-ESTs and the human orthologs for the genes in the PoP set obtained with the human proteome data (c.f. Table 2).

^c ^Relative to the results using the human proteome data.

In summary, only 2 out of 74 core-orthologs were wrongly extended with EST sequences. Also, on the EST level only 111 ESTs were assigned to the wrong ortholog cluster. Thus, the false positive rate was in both cases very low (~3%).

**Figure 2:**
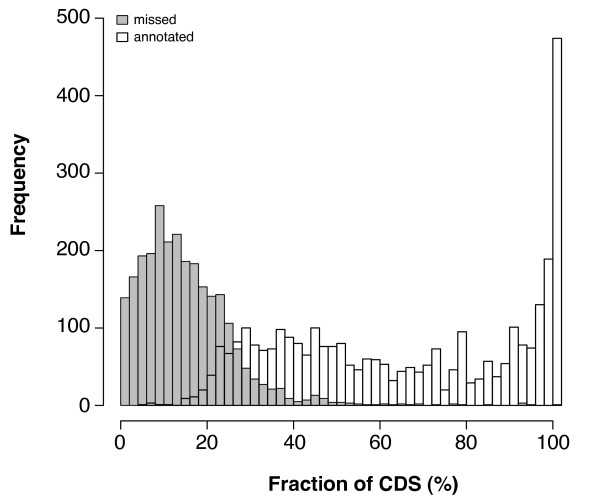
**Sensitivity of HaMStR as a function of CDS coverage**. Fraction of the coding sequence (CDS) covered by the ESTs that have been correctly annotated and missed by HaMStR, respectively.

The sensitivity for detecting an ortholog was also good. From the 81 orthologs present on human chromosome 2 and represented by at least 1 hchr2-EST, HaMStR identified 72 (89%). On the level of individual EST sequences, however, the sensitivity was substantially reduced. The 72 genes for which HaMStR predicted orthologs correctly are represented by 5,899 hchr2-ESTs. HaMStR detected only 3,243 (55%) of these. The ESTs, that HaMStR failed to assign, covered on average less than 13% of the coding sequence of the corresponding gene. Thus, the encoded protein fragment is too short to result in a significant hit in the HaMStR search. Figure 2 displays the dependency of the search result on the fraction of the coding sequence (CDS) covered by an EST. The length distribution of the missed ESTs explains why 9 core-orthologs represented by 389 hchr2-ESTs have been missed entirely. For these ESTs the average coding sequence coverage was in the range of 13%.

Some ESTs, however, have not been assigned even though the encoded protein fragment should be of sufficient length (Figure 2). A visual inspection of a subset of these ESTs indicates that most of them comprise splice variants lacking one or several exons (data not shown).

In summary, the false positive rate of HaMStR is convincingly small and the sensitivity depends strongly on the fraction of the CDS covered by an EST.

To increase the sensitivity of HaMStR, we re-designed the pHMMs such that local alignments between sequence and pHMM are generated during the search (options to the *hmmbuild *command). Using HaMStR with the modified pHMMs, it assigned 5,796 ESTs correctly to all 81 core-orthologs. Compared to the search with the default pHMM structures, this indeed results in a substantially higher sensitivity both on the level of detected genes and ESTs (100% and 92%, respectively). However, 256 ESTs were incorrectly assigned to 11 core-orthologs for which the human ortholog is not located on human chromosome 2. Thus, the gain in sensitivity leads to a pronounced increase of false positive predictions (12%).

### Proof of principle: An EST based phylogeny of Fungi

We have shown that HaMStR efficiently extends core-orthologs with protein or translated EST sequences from further taxa. Now we explore the applicability of the HaMStR approach for large-scale phylogenomic analyses. To this end we analyzed the proteome from 16 completely sequenced fungi and EST data from further 18 fungi (c.f. Table 1). For taxa where ESTs and an annotated proteome are available, we use the ESTs. The collection of 35 taxa, including yeast, is a subset of the taxon set in Fitzpatrick et al. (2006) [3].

HaMStR with *H. sapiens, C. intestinalis, D. melanogaster, C. elegans*, and *S. cerevisiae *as primer taxa was used to compile the sets of orthologs. *S. cerevisiae *was used as the reference-taxon. Please note that we replaced *M. domestica *that was used in the PoP set with *H. sapiens*, since the human genome sequence is considered finished. After running HaMStR with 1,031 core-orthologs and the data from 34 fungi, we obtained a set of extended core-orthologs. For completely sequenced taxa orthologs to almost all genes in the core-ortholog set were found (min: 933 (*Y. lipolytica*), max: 999 (*K. lactis*), mean: 973). For taxa represented by only a few ESTs, the number of detected orthologs ranges from 112 (*T. asperellum*) to 953 (*A. nidulans*) with a mean of 453. Thus, we have an incomplete taxa-gene matrix.

**Figure 3:**
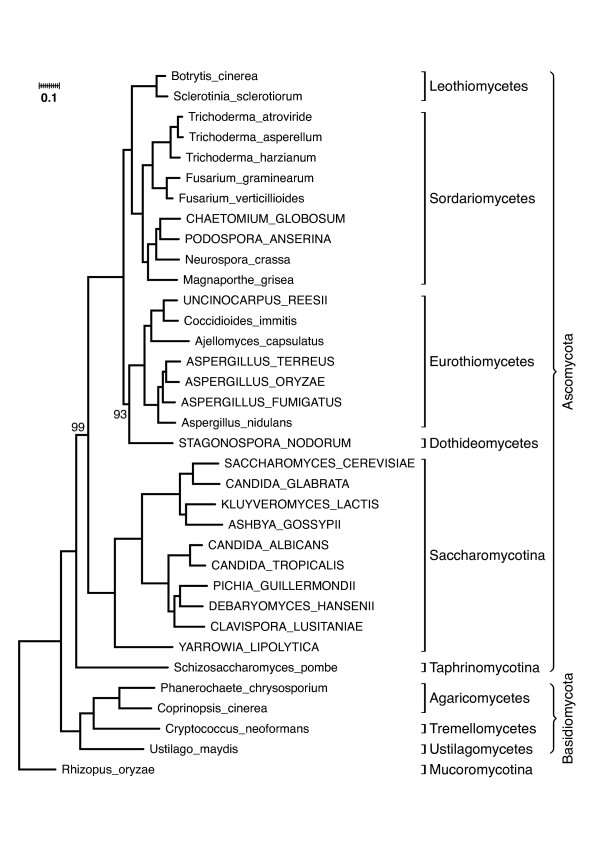
**A maximum likelihood phylogeny of 35 fungi based on 178 genes**. Unless otherwise stated, all splits in the tree have bootstrap support values of 100. For taxa in all upper case letters the annotated proteome was used. For the remaining taxa orthologs were predicted from ESTs.

For the subsequent maximum likelihood tree reconstruction we used 178 genes. Each gene represents at least 40% of the taxa and at the same time each taxon is represented by at least 60% of these genes. The resulting tree has excellent branch support (Figure 3) and is by and large congruent to the phylogeny of these taxa, that was inferred from an analysis of their entire genomes [3]. Only two taxa are differently placed. *Staganospora nodorum*, a dothideomycete, is placed as a sister taxon to the Eurotiomycetes, whereas it is sister to the Sordariomycetes/Leothiomycetes clade in Fitzpatrick et al. (2006) [3]. *Aspergillus nidulans *is positioned basal to the other *Aspergillus *species instead of grouping together with *A. fumigatus*. We cannot rule out the possibility that the differences might be due to erroneous orthology predictions by HaMStR. However, the following explanations argue against a wrong prediction.

For *S. nodorum*, the entire annotated proteome was used. As exemplified in our validation of HaMStR (c.f. Table 2), errors in the orthology prediction are unlikely for entire proteomes. The discrepancy between both trees may be caused by the fact that *S. nodorum *is the only representative of the Dothideomycetes. Together with the short internal branches in this region of the trees an accurate placement of this single taxon is difficult [29]. Moreover, as of today, the Fungi section of the ITOL-project  considers the placement of Dothideomycetes as still unresolved.

*A. nidulans*, the second problematic taxon, is the only *Aspergillus *species in our data for which orthologs are derived from ESTs. No quality information was available for these sequences, indicating that sequencing errors could interfere with the correct placement of *A. nidulans *in our phylogenetic tree. However, our *A. nidulans *placement agrees with other results [30].

Our example clearly shows that HaMStR is well suited to compile phylogenomic datasets, including incomplete sequence data like ESTs or unfinished whole genome sequencing projects.

### Implementation

We offer the HaMStR tool online and for download at . Currently, the users can search EST data for orthologs to core-orthologs derived from six predefined sets of primer-taxa, covering different regions of the metazoan tree. The reference-taxon for the re-BLAST can be selected from the taxa that constitute the primer-taxa. To increase EST data quality, we provide options to clean and cluster EST data, taking into account user information about base qualities [31] of the uploaded sequences. The output gives information to which core-ortholog set a particular EST was assigned to. Links to detailed information about the protein of the chosen reference taxon for the re-BLAST are also provided. A fasta-file containing the sequences of the proteins in the core-ortholog cluster together with a tentative translation of the annotated EST sequence can be downloaded. An example-output is provided on the HaMStR web pages.

## Discussion

ESTs constitute a paradigm of a random sample of fragments from a species' gene set. We show that HaMStR is capable of automatically predicting orthologs for a pre-defined set of genes (core-orthologs) in such data. The results are consistent with orthology predictions obtained with reciprocal BLAST based orthology prediction tools, which however require completely sequenced and annotated genomes. HaMStR performs a targeted search to extend predefined ortholog clusters derived from the analysis of complete genome sequences. This approach has three major advantages.

First, we search for orthologs only to genes for which whole genome comparisons provide prior evidence that orthologs can be identified. For example, core-orthologs including sequences from fungi and animals represent evolutionary old genes that were already present in the last common ancestor of both groups. Thus, an ortholog search for these genes in incomplete and fragmentary data, such as ESTs, from fungi or animals has a good chance of success. In the other extreme, genes with predicted orthologs confined to *e.g*. vertebrates are presumably of evolutionary recent origin or have diverged beyond recognition in taxa outside the vertebrates. Thus, a search for orthologs in ESTs from non-vertebrates will probably fail.

Second, the pHMMs constructed from the core-orthologs allow for a more refined ortholog search compared to a conventional BLAST. In our case example with the human ESTs HaMStR has a very low fraction of false positives (3%, c.f. Table 3). The low fraction of false positive orthology predictions by HaMStR comes at the price of low sensitivity. This is due to the fact that we try to match the pHMM over its entire length to the individual query sequences. ESTs, however, are generally short and cover in most cases only a fraction of the coding sequence. Our search may, therefore, be too stringent. Clustering ESTs prior to the HaMStR search is an obvious strategy to address this problem. However, especially in small EST projects many genes will have only a small fraction of their CDS covered by ESTs, even after clustering. Performing local alignments between sequence and pHMM substantially increases the sensitivity both on the level of detected genes and ESTs (100% and 92%, respectively). Thus, the gain in sensitivity leads to a pronounced increase of false positive predictions (12%). Still, this false positive rate is substantially smaller compared to that of other orthology prediction programs based on a reciprocal best BLAST hit when used with ESTs (data not shown). However, this emphasizes that short local similarities between sequences make orthology inference hard. Most studies -particularly in molecular systematics- rely on an accurate orthology prediction and results can be severely compromised by false positives in the data set. It is a major advance of HaMStR that it automatically filters sequences for which the orthology prediction is unreliable and we suggest using the global pHMM search as default.

Third, HaMStR is computationally efficient. The use of core-orthologs limits the search to only those genes where we expect to find orthologs. Moreover, also targeted ortholog searches are possible.

The performance of HaMStR depends on the choice of the primer-taxa and on the method to determine the core-orthologs. Primer-taxa should cover the diversity of the taxa of interest and thus constitute a scaffold of the corresponding phylogenetic tree. To address the evolutionary relationships of 35 fungal taxa we have used *H. sapiens*, *C. intestinalis*, *D. melanogaster*, *C. elegans*, and *S. cerevisiae *as primer-taxa. In principle, a primer taxon set including several fungi is more appropriate. It would minimize the evolutionary distance to the taxon in which the HaMStR search will be performed. It would also allow searching for orthologs to genes that emerged on the fungal lineage or got lost in metazoa. However, we deliberately did not adapt the primer-taxa. By doing so, we addressed the question of how phylogenomic datasets compiled with HaMStR perform, when no or only a single completely sequenced genome exists for a taxon group of interest. The reconstructed tree for the 35 fungi is entirely resolved, highly supported and compatible to other published phylogenies [3,30]. This indicates that HaMStR, together with this primer-taxon set serves in general as a good starting point for any phylogenomic study within fungi and animals.

For building and training of the pHMMs, a large number of diverse primer-taxa is favorable. However, this holds only if the annotation of genes in the individual genomes is comprehensive. Despite the small set of primer-taxa, each core-ortholog was extended by at least one fungal sequence. Thus, the considerably small set of sequences used to train the pHMMs appears not to impair the sensitivity of the ortholog search in this particular case.

We determined the core-orthologs with InParanoid-TC (see Additional file 1). However, HaMStR allows the use of core-orthologs determined with any orthology prediction method, e.g., orthoMCL [18]. By that HaMStR can be adapted to the particular evolutionary problem one is interested in. If the HaMStR output serves as a data basis for a standard phylogenomics study, many approaches require that each taxon should be represented only by one sequence per gene. In such cases core-orthologs determined with InParanoid-TC or the recently developed OMA-algorithm [32] are a good starting point for HaMStR. On the other hand, if one is interested in the evolution of a gene family, it may be desirable that the ortholog cluster used by HaMStR also contains co-orthologs. In such cases ortholog groups from more inclusive orthology prediction programs can be used as core-orthologs.

## Conclusion

HaMStR extends in a standardized manner a pre-defined set of orthologs with proteins or ESTs from further taxa. A profile Hidden Markov Model that captures the essential features of the known orthologs is used in the ortholog search. The introduction of a reciprocity criterion into the ortholog search makes the orthology predictions symmetric, as is the case when complete proteomes are compared. Variation of the pHMM structure allows searches at different stringency. One may either carry out an alignment of part of the EST- or protein sequence against the entire profile (stringent), or a local alignment of the sequence against part of the profile (relaxed). Profile HMMs used in the HaMStR search can be based on arbitrary primer-taxa and any source of initial orthology predictions. Thus, our procedure is applicable regardless of the molecular systematics problem one is interested in. Testing the performance of HaMStR on a test set of human EST sequences revealed a remarkably low false positive rate of the orthology predictions with good sensitivity. The reconstruction of a phylogenetic tree for 35 fungal taxa based on 178 genes shows that HaMStR can compile informative data sets for contemporary phylogenomic studies. Our procedure may help to standardize data acquisition for studies in molecular systematics, allowing for a better comparability between individual results.

## Methods

For phylogeny reconstruction we aligned the ortholog cluster individually with MAFFT --*maxiterate *1000 --*localpair *[22]. The alignments were concatenated using the perl script *concatenate_alignments.pl*. Alignment columns containing more than 50% undetermined amino acid positions or gaps were removed with the perl script *degapper.pl*. Both scripts are available upon request. Maximum likelihood phylogeny reconstruction was performed with RAxML [33]. The consensus tree from 100 bootstrap replicates was computed with TREE-PUZZLE [34] and the percentages of bootstrap trees supporting a split in the consensus tree were used as branch support values. All datasets compiled in this study are available upon request.

## Authors' contributions

IE designed the study, wrote the software and performed all analyses. SST compiled the data and participated in the data analysis. IE and AvH wrote the manuscript and all three authors read and approved the final version of the manuscript.

## Supplementary Material

Additional file 1 **InParanoid-TC**. Detailed description of Inparanoid-TC. Click here for file

Additional file 2 **Lower ranking HaMStR hits**. Description of the lower ranking HaMStR hits. Click here for file
