# Supplementary material for: HaMStR: Profile hidden markov model based search for orthologs in ESTs
Source: BMC Evol Biol. 2009 Jul 8;9:157. doi: 10.1186/1471-2148-9-157 (PMC2723089; doi:10.1186/1471-2148-9-157)
Supplement: Additional file 1 — InParanoid-TC. Detailed description of Inparanoid-TC. [file 1471-2148-9-157-S1.pdf]

Supplementary Results

Generation of core-orthologs: InParanoid-TC

For this study we compute orthologs for each pair of primer-taxa with InParanoid. The pair-wise orthology predictions are subsequently extended to include all primer-taxa by using a criterion of transitive closure (InParanoid-TC). This approach has the advantage of generating ortholog clusters, with only one sequence per taxon. Such clusters can then be directly used for downstream standard approaches of phylogeny reconstruction. In brief, we order the *n* primer-taxa such that taxon *i+1* is the closest relative to taxon *i* in the species tree, for *i = 1, 2, ..., n-1*, where ties are broken randomly. For each protein in taxon *1* we carry out the following loop:

a) We identify the corresponding InParanoid-orthologs in taxon *2*. If more than one co-ortholog exists, we choose the one with the highest InParanoid-score.

b) With the protein from taxon *2* we identify the ortholog with the highest InParanoid-score in taxon *3*. This procedure continues until the ortholog-pair for taxon *n-1* and *n* has been determined.

c) The circle is then closed, by identifying the ortholog to the protein from taxon *n* in taxon *1*.

d) If the two proteins in taxon *1* at the beginning and the end of the round trip are identical, we keep the set of orthologs and call it **core-ortholog**. Else, we discard the proteins.

We end up with a collection of core-orthologs representing genes present in all primer-taxa. In each individual core-ortholog each taxon is represented exactly once.

From the initial ordering of the primer-taxa, it follows that the newly added sequence in each step is an ortholog to all other sequences already in the candidate cluster (Figure S1). The final closure step (c) excludes pathogenic cases resulting from hidden paralogy [1].

To explore the consistency of InParanoid-TC orthology predictions, we compared our results for the PoP primer-taxa set to those obtained with orthoMCL. 96% of the InParanoid-TC core-orthologs are represented as orthoMCL ortholog clusters of 5 species. A further 3% are represented as ortholog clusters of four species. Thus, almost all InParanoid-TC ortholog clusters are recovered with orthoMCL. InParanoid-TC has one clear advantage over other orthology prediction programs. The pair-wise ortholog predictions from InParanoid can be pre-computed once for a set of taxa. From this data core-orthologs can be rapidly generated for any subset of primer-taxa without further computation.

References

1. Scannell DR, Byrne KP, Gordon JL, Wong S, Wolfe KH: **Multiple rounds of speciation associated with reciprocal gene loss in polyploid yeasts**. *Nature* 2006, **440**(7082):341-345.

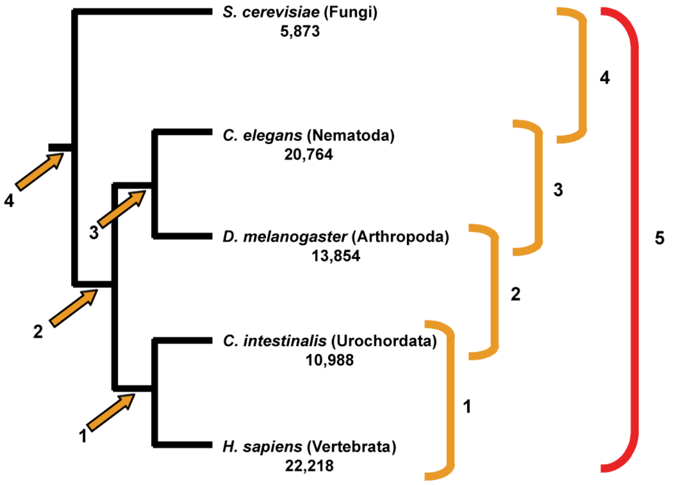

Figure S1 - A phylogenetic justification of InParanoid-TC

InParanoid-TC is an extension of the original InParanoid approach. Two orthologous genes in *C. intestinalis* and *H. sapiens* (bracket 1) are descendents from a single ancestral gene in the most recent common ancestor (MRCA) of the two species (arrow 1). From this it is straightforward to follow that any gene from the more distantly related species *D. melanogaster* that is identified as an ortholog to the *C. intestinalis* gene in the ortholog pair from step 1 (bracket 2) is automatically also orthologous to the corresponding human gene. In the same way it follows that all three genes are descendents from a single gene in the MRCA of *H. sapiens*, *C. intestinalis*, and *D. melanogaster* (arrow 2). The same argumentation applies to the addition of the *C. elegans* and *S. cerevisiae* orthologs (steps 3 and 4). The step 5 closes the circle by confirming that the genes from *S. cerevisiae* and *H. sapiens* are orthologs.
