# Supplementary material for: HaMStR: Profile hidden markov model based search for orthologs in ESTs
Source: BMC Evol Biol. 2009 Jul 8;9:157. doi: 10.1186/1471-2148-9-157 (PMC2723089; doi:10.1186/1471-2148-9-157)
Supplement: Additional file 2 — Lower ranking HaMStR hits. Description of the lower ranking HaMStR hits. [file 1471-2148-9-157-S2.pdf]

Scenario 2, lower ranking HaMStR hits

For 168 core-orthologs more than 1 human protein was assigned as a putative ortholog. For 134 of them InParanoid groups the human proteins as co-orthologs with respect to opossum (23), any other vertebrate (5), *C. intestinalis* (88), or invertebrate species (18). (This implies that the corresponding genes duplicated only considerably recent in evolution). In 8 further cases no InParanoid orthology assignment exists. Comparing their sequences to those of the corresponding HaMStR top hits identifies them as truncated versions of the top-hit proteins. For additional 9 core-orthologs the InParanoid database provided no clear results. For the remaining 16 core-orthologs HaMStR suggested a false positive. In these instances, one of the HaMStR ortholog candidates represents a different albeit similar gene, with distinct orthologs in four of the five species in PoP. The incorrect annotations of these proteins result from missing a corresponding ortholog in our representation of the opossum proteome, either due to their deletion on the opossum lineage, or due to an incomplete annotation of the opossum genome draft sequence. As a consequence, the re-BLAST against the opossum proteome identifies the paralog as a best hit and relates these proteins to the wrong ortholog cluster. However, in all these cases the correct human ortholog was assigned as the top rank ortholog candidate.
